# Marine fungi in the spotlight: opportunities and challenges for marine fungal natural product discovery and biotechnology

**DOI:** 10.1186/s40694-017-0034-1

**Published:** 2017-08-31

**Authors:** Deniz Tasdemir

**Affiliations:** GEOMAR Centre for Marine Biotechnology (GEOMAR-Biotech), Research Unit Marine Natural Products Chemistry, GEOMAR Helmholtz Centre for Ocean Research Kiel, Am Kiel-Kanal 44, 24106 Kiel, Germany

**Keywords:** Marine fungi, 2nd International conference of marine fungal natural products, MaFNaP_2017, Marine fungal biodiscovery, Marine fungal biotechnology

## Abstract

The marine fungal natural products (MaFNaP) Consortium, a scientific network founded in 2014, aims to fuel systematic research on marine fungi and their secondary metabolites. The 2nd international conference of marine fungal natural products (MaFNaP_2017) that was held in Kiel (Germany) and hosted by GEOMAR Centre for Marine Biotechnology (GEOMAR-Biotech) in June 2017 brought together scientists working all relevant aspects of marine fungi. This conference report highlights the topics discussed in the conference and suggestions for future work on marine fungal compounds. One of the major aims is to attract scientists working on terrestrial fungi in tackling the common bottlenecks and to move marine fungal biodiscovery and biotechnology research forward.

## Background

Since ancient times, fungi have played a significant role in basic biotechnological processes such as baking, brewing and dairy products, and later as cell factories for production of commercially important alcohols, enzymes or organic acids. Following the discovery of penicillin in the twentieth century, fungi have become a very important source of pharmaceutical leads developed into life-saving drugs, such as antibiotics and anticancer agents [1]. Terrestrial fungi have been the mainstream of all these efforts, while fungi living in marine world have remained little studied or explored for their potential as sources of natural products or uses in biotechnology.

Fungi are part of marine microbiological communities and found in all habitats as saprotrophs, parasites or symbionts. There is a growing number of reports on their key ecological and evolutionary roles including complex interactions with their hosts and other microbial communities [2]. Many of these roles and interactions are mediated by or linked to their small-molecule natural products. Studies on marine fungi continue to rise with 223 new compounds reported in 2013 and 318 reported in 2014 [3]. This compared to 1992, when only 15 new compounds were reported from marine fungi [4]. Over 100 new fungal metabolites with various biological activities have been reported from phylogenetically diverse marine fungi. Marine fungal natural products lend themselves to large-scale biotechnological cultivation, presenting excellent opportunities in many areas, such as pharmaceuticals or cosmetics, or in food processing and agriculture [5]. Cephalosporin C, a second-line antibiotic was obtained from a *Cephalosporium* (now *Acremonium*) sp. obtained off the Sardinian coast [6]. Plinabulin, a synthetic analog of the diketopiperazine halimide discovered from a marine *Aspergillus* sp. is undergoing late stage cancer clinical trials [7]. However, the systematic evaluation of marine fungi and their secondary metabolites is just starting. Hence there is a great need and prospect for more comprehensive and integrated research, at both basic and applied level, on marine fungi.

## The second international conference of marine fungal natural products (MaFNaP_2017)

The 2nd international conference of marine fungal natural products (MaFNaP_2017) organized by GEOMAR Centre for Marine Biotechnology (GEOMAR-Biotech)/Research Unit Marine Natural Products Chemistry (GEOMAR Helmholtz Centre for Ocean Research Kiel) was held in Kiel, Germany, between 27–29 June 2017 (for more information see [8]). The MaFNaP conferences and the underlying initiative for more systematic research on marine fungal natural products go back to the meeting of a small group of scientists in Prince Edward Island (Canada) in 2014. The MaFNaP Consortium [9] was founded as a scientific network following this meeting. The inaugural MaFNaP conference was held in 2015, organized by the “Mer, Molécules, Santé – MMS” (Sea, Molecules, Health) laboratory and hosted by the Faculty of Pharmacy of the University of Nantes (France). The meeting gathered many scientists for the first time and formalized research efforts and priorities.

The 2nd MaFNaP conference in Kiel substantially intensified the scientific exchange initiated at the first MaFNaP conference. It brought together scientists and stakeholders in order to (i) stimulate and coordinate more systematic research on marine fungi and their secondary metabolites, (ii) stimulate networking and international collaborations for intensive and innovative research, (iii) seek joint funding possibilities, (iv) to increase visibility and pave the long way for revealing the real capacity of marine fungi and marine fungal natural products.

The MaFNaP_2017 conference gathered about 70 scientists representing 13 nations from four continents. The conference featured outstanding investigators from academia and industry, complemented by statements from politics and society. The conference comprised nine plenary lectures as well as 16 short lectures and many flash and poster presentations mainly given by young scientists. A round table discussion focused on future events and research directions. The MaFNaP_2017 assembled an interesting and diverse program on seven topics that are relevant for marine fungal natural products research. The speakers were internationally distinguished scientists: Russell Kerr (Canada, biodiversity and fungal chemodiversity), Robert Capon (Australia, marine fungal chemodiversity), Anake Kijjoa (Portugal, marine fungal chemodiversity), Frank Kempken (Germany, genetics and genomics of marine fungi), Catherine Roullier (France, marine fungal analytics and metabolomics), Marlis Reich (Germany, ecology and physiology of marine fungi), Nina Gunde-Cimerman (Slovenia, halotolerant and halophilic fungi), Eva Stukenbrock (Germany, evolution and host-microbe interactions), Johannes F. Imhoff (Germany, marine biodiscovery) and Vera Meyer (Germany, fungal biotechnology).

The conference confirmed that marine fungi are promising and prolific sources of secondary metabolites. High biodiversity levels of marine fungi associated with many types of marine organisms, ranging from seaweeds, sea grasses, mangroves, sponges, crinoids, corals, crustaceans, fish/shellfish to marine sediments were presented. Intriguing studies were described on mycoviruses and marine fungus-microalgae interactions. Very diverse environments, e.g. tropical, temperate zones including brackish waters (Baltic Sea) and extreme (deep, very cold Arctic) environments have been explored for new cultures of fungi for initial chemical and biological screenings and in-depth chemical work-up. Numerous classes of marine fungal natural products with enormous chemical space and wide ranging pharmacological activities were discussed.

The delegates agreed that cultivability is one of the main obstacles and limitations in biodiscovery and biotechnology of marine fungi. The problem is not specific to marine fungi but applies to all (marine) microorganisms, as part of the so-called “oceans’ dark matter.” Several presentations highlighted successful application of promising techniques involving OSMAC (One-Strain-Many-Compounds) [10] and controlled miniaturization systems on chips [11] to address this issue. Another major problem in (marine) fungal natural product research is that the biosynthetic gene clusters (BGCs) largely remain silent under laboratory culture conditions. This renders the true chemical potential of fungi being mostly inaccessible and leading to rediscovery of known compounds over and over again. Several studies highlighted enhanced chemical diversity, and thereby enhanced biological activity, from marine fungi through OSMAC approach. Another major research focus was the induction of chemical interactions and communication between marine fungi and bacteria in co-cultures, as driver to induce silent BGCs. Many metabolites were reported specifically and only from co-cultures, including the biotransformation of some metabolites by the other co-culture partner [12]. Such approaches were presented in more than a quarter of the communications indicating a real trend in the MaFNaP community. Additional approaches to control/improve the natural product biosynthesis e.g. chemical cues (epigenetic modifiers) or physical cues (heat shock) were also described.

In recent years, revolutionary metabolomics approaches have significantly improved the chemical profiling and dereplication (identification of the known metabolites mainly by LC–MS by comparison of their molecular weight/molecular formulae against in-house/commercial databases) of natural products, including those from marine fungi. Novel methods such as molecular networking using GNPS platform and in silico MS/MS databases reveal the real chemical inventory of marine fungi. They are not only useful in identifying the known members of many molecular clusters, but also their new derivatives, and even new clusters at the extract stage. In addition, marine fungal chemists have reported the development of new, automated platforms/tools for detecting specific types of compounds/substituents, e.g. halogenated marine fungal metabolites. Subsequent purification studies led to the isolation of new halogenated compounds. Targeted and untargeted metabolomics approaches associated with time course analyses shed light on dynamics of fungal metabolome, growth cycle and biosynthetic pathways.

Other discussed topics included the genomics and transcriptomics of marine fungi, gene regulation/chromosome stability, ecology, taxonomy/identification, halotolerance and halophily. These are crucial for understanding the roles of fungal natural products in Nature and for overcoming the existing bottlenecks to realize their true application potential in many areas.

Excellent lectures highlighted the importance of marine fungi in biodiscovery especially in targeting human cancers, microbial infections and many other diseases. Finally, the newest developments in biotechnology of fungi were discussed including synthetic biology tools. These tools are applicable to marine fungi and crucial for exploring and exploiting their potential in biotechnology and large scale production.

The MaFNaP_2017 conference was supported by the Kiel Cluster of Excellence “The Future Ocean” [13] and the Ministry of Economic Affairs, Employment, Transport and Technology of Schleswig Holstein (Germany). The conference collaborated with the European Society of Marine Biotechnology [14]. Further support was received from industry, publishers and journals, indicating the relevance of the topic for research and development. The conference provided several travel grants and best poster/short lecture awards to young researchers.

## Conclusions and future outlook

The second MaFNaP conference was in many ways a success, bringing very diverse expertise together to maximize the opportunities to advance the work on marine fungal metabolites and enabling collaborations among scientists. The meeting confirmed substantial progress on metabolomics. However more efforts and collaborations in all omics—genomics, transcriptomics and proteomics—should be integrated into metabolomics work already in the earliest stages of biodiscovery to guide the subsequent work.

Besides the classical approaches, the delegates agree that ecology should also be incorporated into drug discovery efforts. Ecological approaches (e.g. quorum sensing) are powerful in identification of bioactive compounds. Future work should also involve marine fungal communities and investigation of their interactions with their host and the complex microbial networks they live in. The in-depth understanding of the chemical language they speak will lead to discovery of natural products with beneficial functions. The MaFNaP community envisions that in the near future innovative techniques will address the issue of cultivability. On the other hand innovative biotechnological approaches and efficient heterologous expression systems will be needed for full exploration of cultivable and non-cultivable marine fungi in all stages of the discovery-to-development pipeline. This knowledge is of interest to all fungal scientists.

The MaFNaP meetings will continue as an interactive, informal and biannual conference. The 3rd MaFNaP conference is planned to take place in Athens, Greece, in 2019. The MaFNaP Consortium invites and encourages scientists working on all relevant aspects of (marine) fungi for engagement and interactions.

Look forward to meeting many of you at the MaFNaP_2019 conference!

**Figure:**
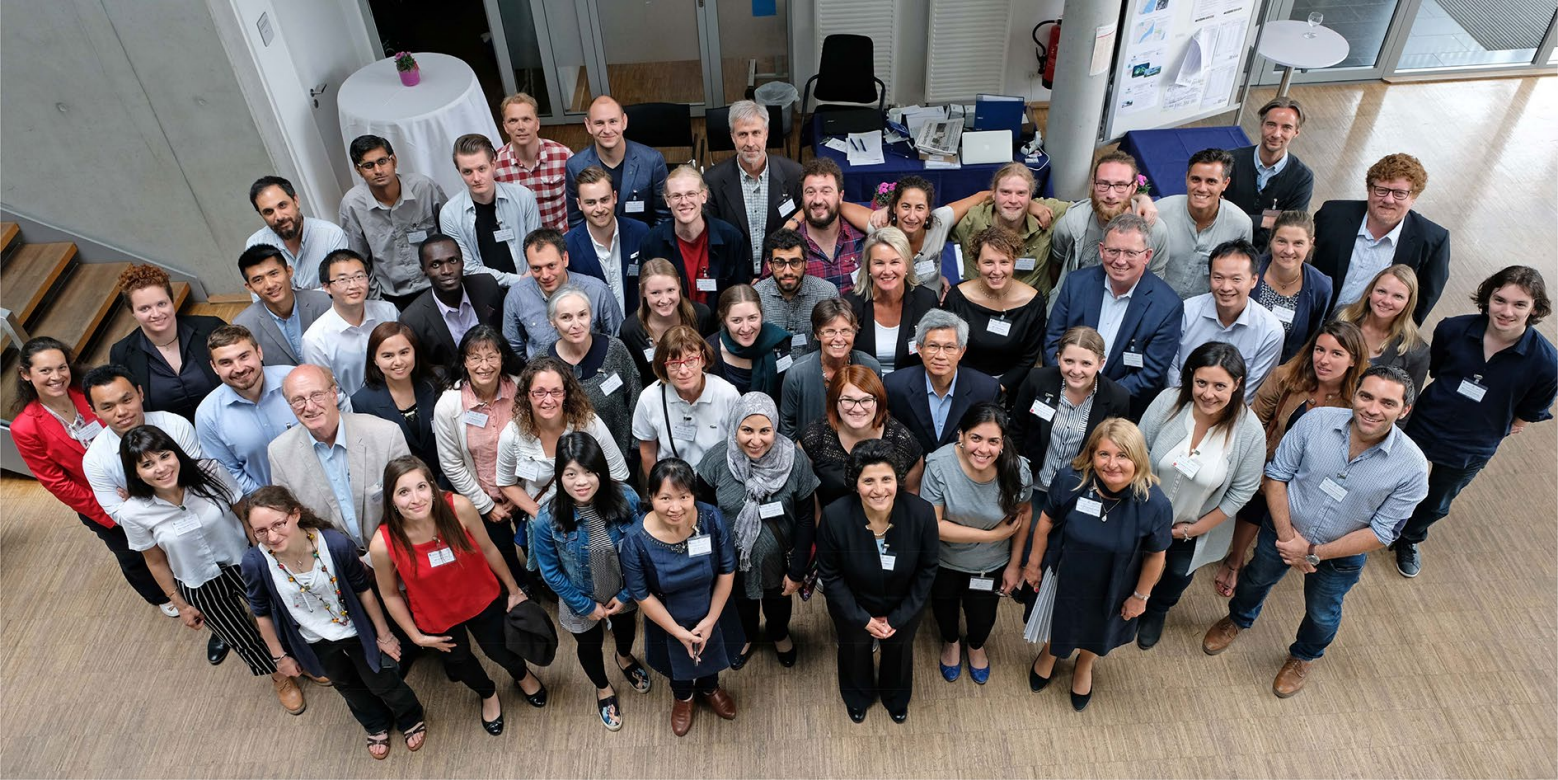
Photo: Jan Steffens, GEOMAR

## Abbreviations

MaFNaP: marine fungal natural product
BGC: biosynthetic gene clusters
GNPS: global natural product social molecular networking
LC–MS: liquid chromatography coupled to mass spectrometry
